# Characterizing the Intrinsic Background in XPS Using the Narrow-Shirley Approach

**DOI:** 10.1021/acs.jpcc.5c07020

**Published:** 2025-12-15

**Authors:** Alberto Herrera-Gomez, Dulce Guzman-Bucio, Dagoberto Cabrera-German, Antony D. Dutoi, Milton Oswaldo Vazquez-Lepe, Orlando Cortazar-Martinez, Abraham Jorge Carmona-Carmona

**Affiliations:** † 428213 CINVESTAV-Unidad Queretaro, Cinvestav 76230, Queretaro, Mexico; ‡ Departamento de Investigación en Polímeros y Materiales, 27813 Universidad de Sonora, Hermosillo 83000, Mexico; § Department of Chemistry, 7699 University of the Pacific, Stockton 95209, California, United States; ∥ Departamento de Ingeniería de Proyectos, 42566 Universidad de Guadalajara, Guadalajara 44430, Mexico; ⊥ 3972 Benemérita Universidad Autónoma de Puebla, Puebla 72000, Mexico

## Abstract

The background signal in X-ray Photoelectron Spectroscopy (XPS) consists of extrinsic and intrinsic components. This study focuses on characterizing the intrinsic background, particularly its structure across an extended binding energy (BE) range beyond the vicinity of the main photoelectron peak. The structure of the intrinsic background in the extended region can be reproduced through a rich set of wide Gaussian peaks. In the near-peak region, the intrinsic background is successfully characterized using the empirical Shirley algorithm. Since the Shirley algorithm fails when applied to extended energy regions by overshooting the experimental signal, a modified version must be employed that decays beyond the near-peak region. We found that a functional form flat near the peak (as in the Shirley algorithm) decays in a Gaussian-like manner for higher binding energies, satisfactorily reproduces the experimental data, and allows for revealing the rest of the rich structure of the intrinsic background. For this reason, we termed it a narrow-Shirley (NS) background. The characterization of the structure of the extended region of the intrinsic background enables the qualitative exploration of its physical origin. Building on previous work linking the near-peak Shirley signal to Interchannel Coupling with Valence Band Losses (ICVBL), we propose and provide qualitative evidence that this ICVBL mechanism is responsible for the entire intrinsic background structure across the extended energy range; this mechanism involves the absorption of a photon by a participating core level. Two of the predictions of the ICVBL mechanism are tested by comparing the structure of the intrinsic background with Auger Electron Spectroscopy (AES) and X-ray Absorption Spectroscopy (XAS) data; a third prediction, the expected modulation of the intrinsic background with photon energies around the threshold of the participating core level, is tested through synchrotron experiments.

## Graphical abstract

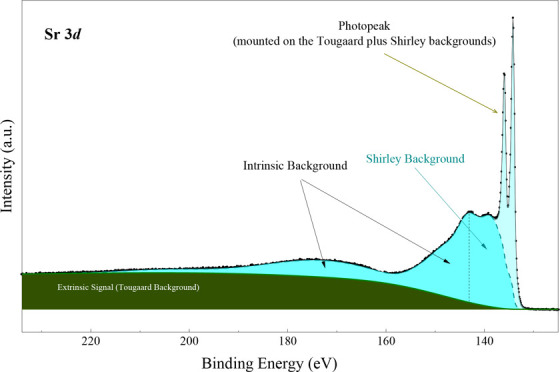

## 1 Introduction

### 1.1 The Need for an Alternative to the Traditional Shirley Algorithm

Accurate background subtraction is a prerequisite for reliable quantitative analysis in X-ray Photoelectron Spectroscopy (XPS). Historically, the Shirley background has been widely adopted and, within a limited energy window, is an effective empirical approximation of the intrinsic background in the near-peak region. However, a common misconception exists among the wider XPS community, where the Shirley function is often broadly cited as covering inelastically scattered electrons, failing to distinguish between extrinsic and intrinsic background components. Correcting this thinking is essential for understanding the fundamental limitations in spectral analysis.

While the traditional Shirley background performs adequately for narrow-scan spectra, its empirical formulation fails to accurately describe the intrinsic background components that manifest over wider energy ranges. As shown in spectra covering extended binding energy (BE) regions (e.g., Figure 1b), the intrinsic background structure on the higher BE side of the main peak does not simply level off as implied by the conventional model; instead, it often displays significant complexity and then drops at even higher BE values. When applied to these extended windows, the traditional Shirley function tends to overestimate the experimental background signal, introducing inaccuracies in the determination of the peak area.

**Figure 1:**
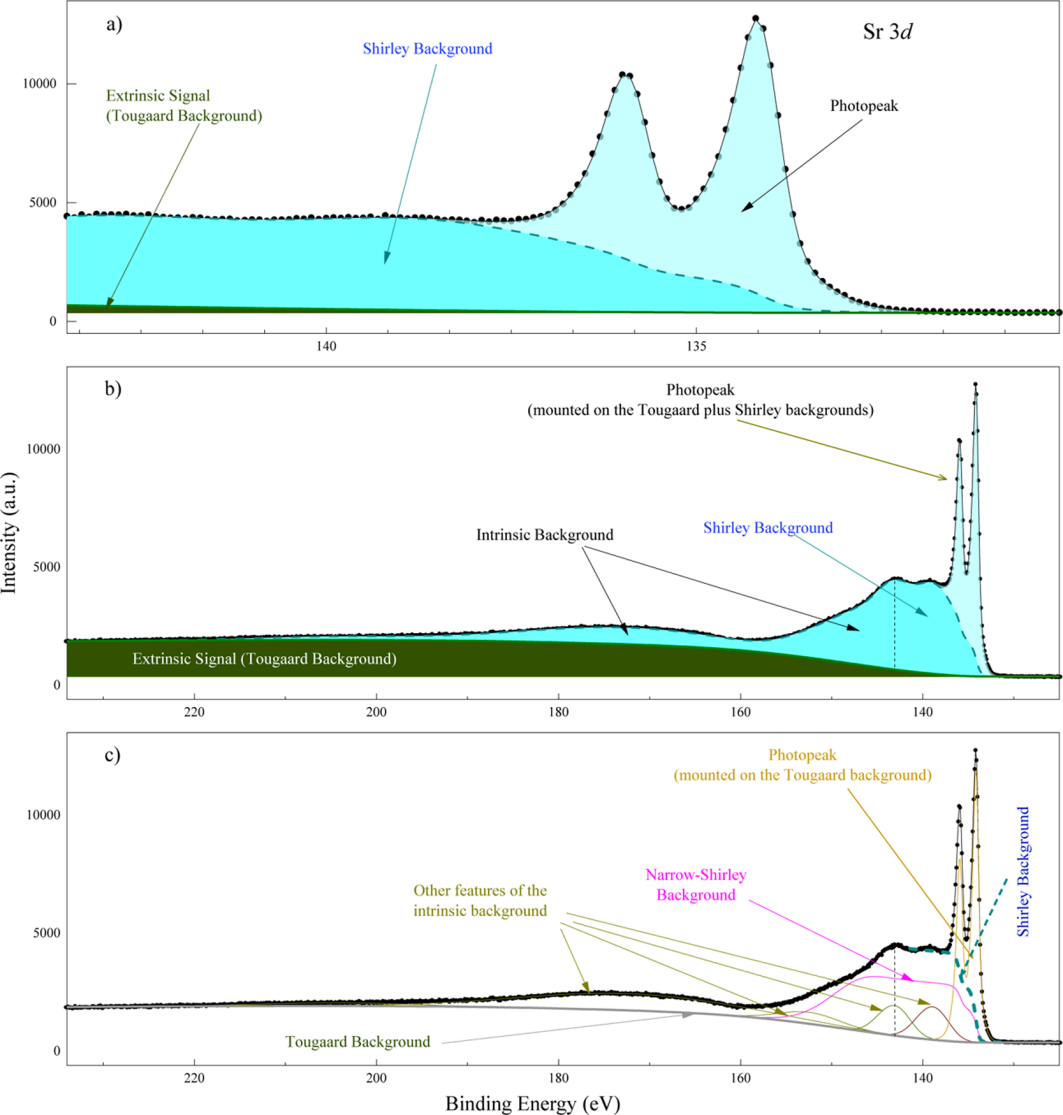
Sr 3*d* spectrum acquired with an Al Kα X-ray source. (a) Near-peak region that is usually employed for peak-fitting analysis. The Shirley background (darker shade of blue) dominates over the peak (light blue) and Tougaard background (green). (b) The vertical dashed line demarks the left limit of the plot in (a), showing that the Shirley background is part of the intrinsic background. The intensity of the Tougaard background dominates in wider energy ranges. The area associated with the Shirley background in (a) extends nontrivially toward higher binding energy and no longer resembles a step function. (c) At higher binding energies, the intrinsic signal reveals a rich structure (labeled as “other features of the intrinsic background”). It is important to note that these peaks do not correspond to photoelectron signals of their satellites (the peak-fitting parameters are indicated in Table 1).

This limitation necessitates a refinement of the approach. We have thus developed the narrow-Shirley (NS) background model to accurately characterize this intrinsic background behavior across wider BE ranges. The NS model is specifically designed to follow the established, effective form of the traditional Shirley model in the near-peak region—thereby utilizing its proven success in that small window—but it introduces a decay mechanism that causes the function to roll off and drop further away from the peak’s maximum. This functional modification provides a more realistic representation of the energy distribution of the intrinsic background signal across extended BE ranges, ensuring a superior fit and facilitating a more robust analysis of other intrinsic spectral features (such as satellites) and the final quantitative results.

The precise subtraction of the background signal is a fundamental requirement for achieving accurate quantitative chemical composition analysis in XPS. The NS background model addresses this challenge by providing a robust and efficient fitting solution. From a fundamental perspective, the NS model allows for significantly better fits by accurately characterizing the intrinsic background structure, which is especially critical when the analysis involves an extended binding energy (BE) range. As demonstrated in the subsequent analysis, the NS background is recommended for routine quantitative analysis when the spectrum’s binding energy range exceeds 15 eV, ensuring both high accuracy and workflow efficiency.

The name “narrow-Shirley” (NS) refers directly to the functional behavior of our model. The term “narrow” is used to describe the behavior of the background function at higher BEs:

- The traditional Shirley background is defined as an integral over the peak area, meaning that its functional form extends indefinitely toward higher BEs.
- In contrast, the narrow-Shirley model is explicitly designed to truncate or decay its contribution beyond the near-peak region.

Therefore, the term “narrow” is used to signify that the background signal does not extend to infinity for higher binding energies. The NS model follows the effective form of the Shirley function in the near-peak region but introduces a necessary decay mechanism that narrows the effective energy range of the background contribution, aligning it with the physical reality of intrinsic inelastic losses in extended spectra. This modification allows the NS model to be reliably applied to wider energy range spectra without the oversubtraction error associated with the indefinitely extending traditional Shirley function.

The NS background model is user-friendly, as its parameters are automatically optimized during peak-fitting.

### 1.2 Physical Significance

XPS is a vital technique for probing the electronic structure of materials by analyzing the energy distribution of the emitted electrons. Accurate interpretation of XPS data necessitates a comprehensive understanding of various signal components, including the background signal.^1^ A crucial distinction must be made between different signal components:

- Intrinsic signal: originates from electrons ejected (e.g., photoelectrons, Auger electrons) that travel through the solid and escape into vacuum without experiencing inelastic scattering. These contribute to distinct spectral features such as peaks.
- Photoemission peaks and satellites: originate from electrons ejected by the photoelectric effect that travel through the solid and escape into vacuum without experiencing inelastic scattering (they correspond to case b in Figure 8).
- Extrinsic signal: arises from electrons (originally from intrinsic processes) that undergo at least one inelastic scattering event while traversing the solid, losing energy and forming a broad background. This component is often modeled using approaches based on the electron energy loss function (EELF), such as the Tougaard^2^ and the partial-intensity^3^ methods.
- Intrinsic background: refers specifically to the portion of the intrinsic signal that forms a background structure, distinct from the main sharp photoelectron, satellites, or Auger peaks. Its origin is not due to extrinsic inelastic scattering of the primary photoelectron being analyzed.^4^ There is a widespread lack of awareness of the intrinsic background existence and a deeply ingrained belief in the XPS community that the entire background is due to inelastically scattered photoelectrons. This perspective incorrectly attributes all background signal to the extrinsic component. It should be noticed that the intrinsic background cannot be modeled through electron-energy-loss-function formalisms (it corresponds to case d in Figure 8).
- Shirley background: conventionally refers to the step-like increase often observed on the high-BE side of photoelectron peaks, particularly in the near-peak region.^5^ It is typically modeled empirically using algorithms like the Proctor–Sherwood method^6^ and is considered part of the intrinsic background.
- Narrow-Shirley (NS) background: the specific, empirical method introduced in this work (Section 3) to quantify the intrinsic background over an extended BE range. It mimics the Shirley behavior near the peak but decays further away, enabling the analysis of other intrinsic background features.

Figure 1 shows an example of these components. Despite the prominence of the Shirley component in the near-peak region, its physical origin remains a subject of debate, as extrinsic scattering frameworks do not adequately explain it.^4^ One of the objectives of this work is to explore the physical origin of the intrinsic background through characterization of its structure. We employ the NS method, detailed in Section 3, to carry out this characterization. The NS method preserves the near-peak step-like behavior while decaying at higher BEs, which enables the identification of additional intrinsic background structures. The Narrow-Shirley algorithm is implemented in freely available software.^7^

We propose that the interchannel coupling with valence band losses (ICVBL) mechanism (Section 4.2) accounts for the entire intrinsic background structure over extended energy ranges, an idea previously linked to the near-peak Shirley signal.^4^ This study explores the nature of this often-overlooked signal component by comparing XPS data to results from Auger electron spectroscopy (AES) and X-ray absorption spectroscopy (XAS). We focus primarily on spectra from pure metals to isolate and understand these fundamental background features before extending the analysis to more complex chemical systems.

## 2 Experimental Details and Data Analysis Methods

### 2.1 Experimental Data Acquisition

#### 2.1.1 XPS Data

Raw spectra were obtained for pure metallic Sr, Pd, and Sc from the XPSLibrary database.^8,9^ The Sr and Pd 3*d* spectra were acquired from 99% pure samples using a Quantum 2000 PHI system with a monochromatic Al Kα source. The samples underwent in situ Ar-ion beam etching before analysis. The pass energy was 23.5 eV, and one scan was recorded per spectrum with dwell times of 2.402 s for Sr 3d and 2.282 s for Pd 3d.

The Sc 2*p* X-ray photoelectron spectrum was acquired using a Surface Science Instruments X-probe system equipped with a monochromatic Al Kα source from 99.99% pure tantalum. A pass energy of 29.7 eV was used in constant analyzer energy (CAE) mode with 45 scans and a dwell time of 0.1 s. The operating pressure was 1.6 × 10^–9^ Torr. Additional experimental details are available in ref 10. All 3*d* and 2*p* spectra were recorded at a takeoff angle of 90°.

An Au 4*p* spectrum was obtained from the database of La Trobe University.^11^ The data correspond to aluminum foil cleaned with 2-propanol and analyzed using a Kratos Axis NOVA system with a monochromatic Al Kα X-ray source. The foil underwent in situ Ar-ion beam etching for 300 s, with 30 s pauses between sets of region scans. Data acquisition was performed using a pass energy of 5 eV, a dwell time of 0.25 s, and 10 scans at a takeoff angle of 90°.

Spectra for the Ni 3*p* and 3*s* levels were obtained from a metallic thin film, with experimental details provided in a previous study.^12^ The data was collected using a spectrometer with a pass energy of 15 eV, a step size of 0.1 eV, a dwell time of 0.2 s over 25 scans, and a takeoff angle of 90°.

A Pb 4*f* spectrum was obtained from ref 13. The high-purity lead sample was sourced from Alfa Aesar. The lead foil was ultrasonically cleaned by using hexane, acetone, and methyl alcohol. The surface was then scraped with a clean razor blade inside a fume hood and mechanically mounted onto a sample holder for analysis.

#### 2.1.2 Auger Data

The KLM Auger spectrum was digitized from a publication by Egri et al.^14^

#### 2.1.3 NEXAFS Data

K and L_2_,_3_ edge X-ray Absorption Near-Edge Structure (NEXAFS) spectra for Sr and Pd were obtained from the Materials Project Database.^15–17^ The crystal structures used were hexagonal (Sr: *P*6_3_/*mmc*) and cubic (Pd: *Fm̅*3*m*). As these represent stable phases that may differ from the XPS samples, the NEXAFS comparison discussed below (Section 4.3.2) is considered qualitative. The energy origin for NEXAFS spectra was set at the first derivative maximum in the edge region.

#### 2.1.4 Synchrotron Data

A Ti film was DC magnetron sputtered onto an RCA-cleaned Si(100) substrate (200 W, 20 sccm Ar, 15 min, and +70 V substrate bias). The film was immediately capped in situ with an ultrathin Al layer by sputtering (100 W, 20 sccm Ar, 3 s, +20 V substrate bias) to prevent oxidation.

HAXPES was performed at the NIST SST-2 beamline (NSLS-II). Photon energies ranged from 4594 to 5400 eV (Si(220) monochromator). The geometry was grazing incidence (5° to surface) with a fixed 80° takeoff angle. Spectra were acquired using a 400 mm diameter concentric hemispherical analyzer with a 200 eV pass energy, 0.100 s dwell time, 0.2 eV step size, and 10 scans.

### 2.2 Data Analysis Methods

To isolate the fundamental structure of the intrinsic background, we selected spectra from pure metallic elements. This approach minimizes the effects of chemical shifts and complex bonding environments typically found in compounds or alloys, allowing for a more accurate characterization of the intrinsic background signal.

All peak-fitting analyses were conducted using the AAnalyzer software^18^ employing a consistent and systematic methodology comprising the following advanced analysis methods:

- Active Background Approach: The background shape and intensity were simultaneously determined along with the peak parameters, rather than being subtracted before fitting. This concurrent modeling allowed for the integration of multiple background contributions, enhancing the accuracy of the fitted parameters.^19^
- Background Modeling: The total background signal was decomposed into two primary components.
  An extrinsic background: This component represents inelastic losses and was modeled using the Tougaard formalism, a well-established method for quantifying extrinsic scattering effects.^2^
  An intrinsic background component: The intrinsic background was modeled using the narrow-Shirley (NS) method (introduced in this paper), a modified version of the Shirley–Vegh–Salvi–Castle (SVSC) approach (the SVSC background is a variant of the Shirley background in which the Shirley background is applied to one peak; a full description is in ref 19. Additional peaks are required to account for the rich structure of the intrinsic background in the extended BE region. Unlike conventional Shirley algorithms, the NS method incorporates a decay function at higher binding energies, enabling the characterization of additional intrinsic features beyond the near-peak region.
- Peak lineshape analysis: asymmetric core-level peaks were fitted using the coupled-resonances (CR) line shape, a theoretically derived model that extends the Lorentzian distribution to account for interference effects.^20^ The CR line shape provides improved fitting accuracy for asymmetric peaks and can be used for chemical composition calculations. Symmetric peaks were fitted using Voigt or Gaussian lineshapes.
- Uncertainty assessment: uncertainties in fitted parameters, including peak areas, widths, binding energies, and background parameters, were quantified using the Covariant Matrix Approach. This method accounts for correlations among all fitting parameters, providing a comprehensive confidence interval assessment.^21,22^

This analytical framework effectively separates the peak signal, extrinsic background, and structured intrinsic background, enabling a detailed analysis of the intrinsic background structure across extended binding energy ranges.

Detailed fitting parameters and comprehensive analysis results for all spectra are available through the XPS Peak-Fitting Network Platform (XPSOasis).^23^

## 3 The Narrow-Shirley Method for Characterizing the Intrinsic Background

As mentioned above, conventional Shirley algorithms are unsuitable for consistently analyzing the intrinsic background over wide energy ranges. To overcome this limitation and enable the characterization of the intrinsic background structure across extended binding energies (BEs), we employed the narrow-Shirley (NS) method. It is essential to emphasize that the NS method is an empirical tool specifically designed to quantify and reveal the structure within the intrinsic background signal over broad ranges; it is not intended as a fundamental physical model in itself. This approach allows for applying a Shirley-like background shape in the near-peak region while ensuring a physically reasonable decay at higher BEs, allowing for the identification and fitting of additional features within the intrinsic background.

### 3.1 Components of the Photoemission Signal and Background Modeling Challenges

#### 3.1.1 The Photoemission Process and Signal Detection

Photoemission involves the ejection of an electron (photoelectron) from a core or valence level when it absorbs a photon with an energy *h*ν greater than its binding energy *E*_B_. The emitted electron carries kinetic energy equivalent to the difference between the photon energy and the binding energy. This primary process results in the formation of a core hole.

While many photoelectrons are generated, only a fraction reaches the detector. During transit through the solid, some electrons lose energy through inelastic scattering, contributing to the extrinsic signal background. Additionally, some electrons may be absorbed by the chamber walls and do not contribute to the detected signal.

Electrons that arrive at the analyzer without energy loss form sharp spectral features, contributing to the intrinsic signal. In contrast, those that experience inelastic scattering form a broad extrinsic background. It is crucial to recognize that other electron emission processes, such as Auger decay and interchannel coupling mechanisms,^24^ also contribute to the overall intrinsic signal, potentially creating additional peaks or background structures.

#### 3.1.2 Visualizing Intrinsic and Extrinsic Contributions

The distinction between intrinsic and extrinsic signals is essential for interpreting the total measured spectrum. This separation is illustrated in Figure 1 using the Sr 3*d* spectrum as an example.^25^ The extrinsic signal, represented by the green area in Figure 1b, arises from inelastic scattering and can be calculated by using electron energy loss function (EELF)-based methods, such as the Tougaard approach. The signal above the thick green line in Figures 1b,c corresponds to the intrinsic signal, including the photoelectron peaks.

Figure 1a focuses on the near-peak region typically analyzed for chemical state determination. In this region, the intrinsic signal is dominated by the main photoelectron peaks (light blue) and the conventional Shirley background (darker blue). Notably, in this limited range, the intensity of the Shirley background significantly exceeds that of the Tougaard background.

Figure 1a creates the illusion that the Shirley background extends indefinitely toward higher BE. However, examining a wider energy range (Figure 1b,c) reveals that the intrinsic signal not only decays but also exhibits a rich structure beyond the near-peak region; this is often overlooked. This extended intrinsic background, which is not adequately accounted for by extrinsic loss models, is the primary focus of this study. While the Tougaard background dominates the spectrum at higher binding energies, the intermediate region contains substantial contributions from structured intrinsic processes that merit detailed characterization. It is important to remark that the peaks labeled as “other features of the intrinsic background” do not correspond to photoemission peaks (or their satellites), nor plasmon features.

#### 3.1.3 Limitations of Standard Background Models for Extended Range Analysis

Accurate spectral analysis requires the appropriate background modeling. While the Tougaard method provides a robust theoretical framework for the extrinsic background in bulk materials, modeling the intrinsic background presents challenges, especially over extended ranges.

The conventional Shirley background, while widely used and visually appropriate for the step-like feature in the near-peak region (Figure 1a), suffers from significant limitations when applied over wider energy ranges. The calculated Shirley background shape and intensity are susceptible to the chosen energy range (end points) used for calculations, potentially leading to unphysical variations as shown in Figure 2. This makes its application unreliable for consistent analysis beyond the near-peak region.

**Figure 2:**
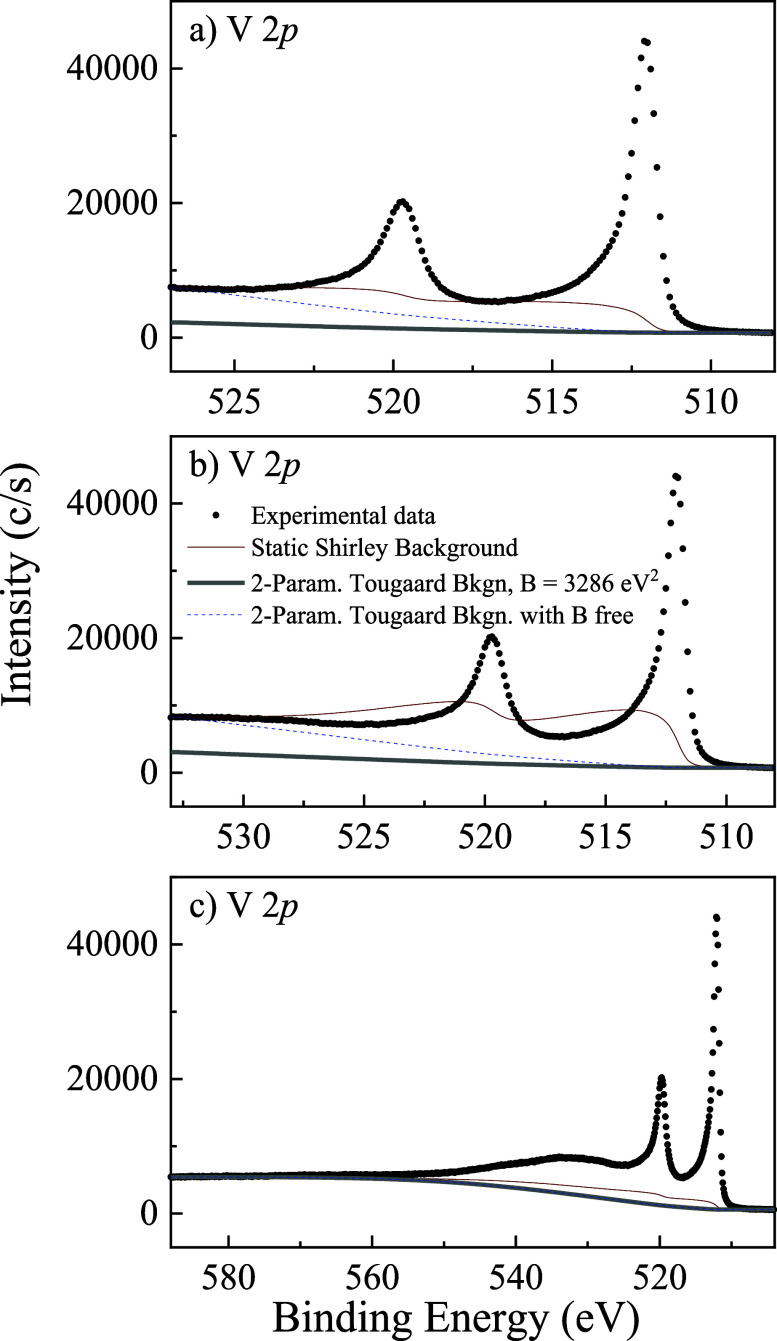
Background modeling for the metallic V 2*p* core level spectrum using different methods across different binding energy ranges. Solid brown lines correspond to the 2-parameter Tougaard background with B fixed to 3286 eV^2^ and C to 1643 eV^2^; red lines to the Shirley background calculated using the Proctor & Sherwood (P&S) method; dashed blue lines to the two-parameter Tougaard background with a value of the B-parameter chosen to match the experimental data in the high binding energy range. (a) In the near-peak range, P&S converges, but the fitted Tougaard deviates from the theoretical curve. (b) Over a broader BE range, P&S fails to converge (after six iterations) and behaves unphysically. (c) At even wider BE ranges, P&S converges but to different values than in (a), while the free B-parameter and theoretical B-parameter Tougaard backgrounds coincide.

The Tougaard model for the extrinsic background is theoretically grounded; however, common errors in its application can occur, such as its inappropriate use in analyzing the background from thin layers or the use of unphysical parameters in the two- or three-parameter Tougaard background to force agreement with the total signal far from the peak (equivalent to ignoring the intrinsic component).

These limitations highlight the need for an alternative approach, such as the Narrow-Shirley method introduced in the next section. This method is specifically designed to empirically characterize the structure of the intrinsic background over extended energy ranges where conventional Shirley methods fail, without conflating it with the physically distinct extrinsic background described by Tougaard.

### 3.2 Functional Form of the Narrow-Shirley Structure

The NS method modifies the Shirley–Vegh–Salvi–Castle (SVSC) algorithm.^19^ Since the latter is essential for the description of the narrow-Shirley structure, a brief description follows.

The SVSC approach associates a Shirley-type background contribution with a specific peak (*i*) in the spectrum, which is calculated as

1 $$B_{SVSC,i}\left(E\right)=\int_{-\infty }^{\infty }dTP_{i}\left(E+T\right)S_{SVSC,i}\left(T\right)$$

where *P*_*i*_(*E* + *T*) is the intensity of the *i*-th peak (excluding its associated background) at energy *E* + *T*, and *S*_SVSC,*i*_(*T*) is the Shirley-like response function for that peak. This response function is expressed using a step function θ as follows:

2 $$S_{SVSC,i}\left(T\right)=s_{SVSC,i}\theta \left(T\right)$$

with *s*_SVSC,*i*_ the associated SVSC parameter;^19^ it quantifies the intensity scaling factor for the Shirley-like step generated by peak *i*. This leads to the standard SVSC background expression:

3 $$B_{SVSC,i}\left(E\right)=s_{SVSC}\int_{-\infty }^{\infty }dTP_{i}\left(E+T\right)\theta \left(T\right)=s_{SVSC}\int_{0}^{\infty }dTP_{i}\left(E+T\right)$$

The key modification in the narrow-Shirley (NS) method is replacing the simple step function *S*_SVSC_(*T*) with a function *S*_N_(*T*) that mimics the step near the peak (*E* ≈ *E*_0,*i*_) but decays toward zero at higher binding energies (far from *E*_0,*i*_). This contrast is illustrated schematically in Figure 3.

**Figure 3:**
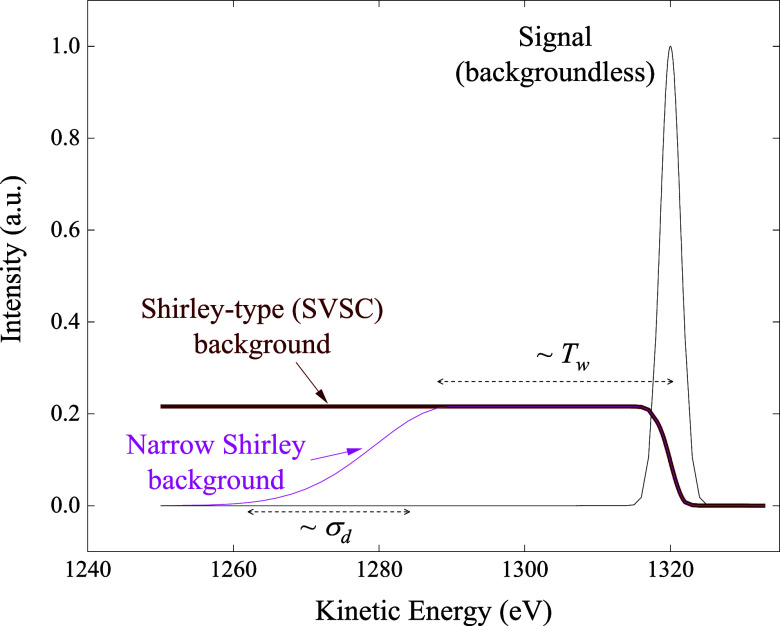
Contrast between traditional Shirley and narrow-Shirley backgrounds. They coincide with each other in the near-peak region. The parameters *T*_w_ and σ_d_ are employed in eq 5.

The resulting NS background structure associated with peak *i* is the following:

4 $$B_{NS}\left(E\right)=\int_{0}^{\infty }dTP_{i}\left(E+T\right)S_{NS}\left(T\right)$$

A practical functional form proposed for the narrow-Shirley response function *S*_N_ is the following:

5 $$S_{NS}\left(T\right)=s_{NS}\times \{\begin{array}{ll} 0 & T<0\\ 1 & 0\leq T\leq T_{w}\\ \exp\left(-{\left(\frac{T-T_{w}}{\sigma_{d}}\right)}^{2}\right) & T_{w}<T \end{array}$$

Here, *T*_w_ (width) defines the approximate energy range beyond *E*_0,*i*_ where the NS function behaves similarly to the standard Shirley step, and σ_d_ (decay) determines the width of the Gaussian-like decay region where the function smoothly transitions toward zero (see Figure 3).

Using the NS method allows for the primary step-like component of the intrinsic background associated with a peak (like the Sr 3*d* peak in Figure 1c) to be effectively modeled over a wide range. Crucially, because the NS function decays, it facilitates the identification and fitting of additional distinct peaks required to fully describe the complex structure of the total intrinsic background at higher binding energies. The three parameters of the NS background (*s*_NS_, *T*_w_, and σ_d_) are optimized during fitting.

### 3.3 Relationship between NS-Resolved Intrinsic Background and Conventional Shirley

When the NS method is applied, the total intrinsic background is modeled by the sum of the NS function (eq 4) and other peaks (e.g., the additional doublets shown in Figure 1c for Sr 3*d*). It is instructive to compare this NS-resolved total intrinsic background to the background calculated by using a conventional Shirley algorithm applied only over the narrow, near-peak region traditionally used for chemical analysis.

As shown in Figure 4b for Au 4*p*, the shape and intensity of the NS-resolved total intrinsic background (i.e., the sum of the NS function and the extra peaks associated with the intrinsic background) in the near-peak region closely match those obtained from a standard Shirley calculation confined to that region. The extrinsic inelastic background of the Au 4*p* photoemission spectrum was fitted using the Tougaard algorithm^2^ by convoluting the total spectrum with the experimentally determined normalized inelastic cross section digitized from ref 26. Fitting parameters are listed in Table 2.

**Figure 4:**
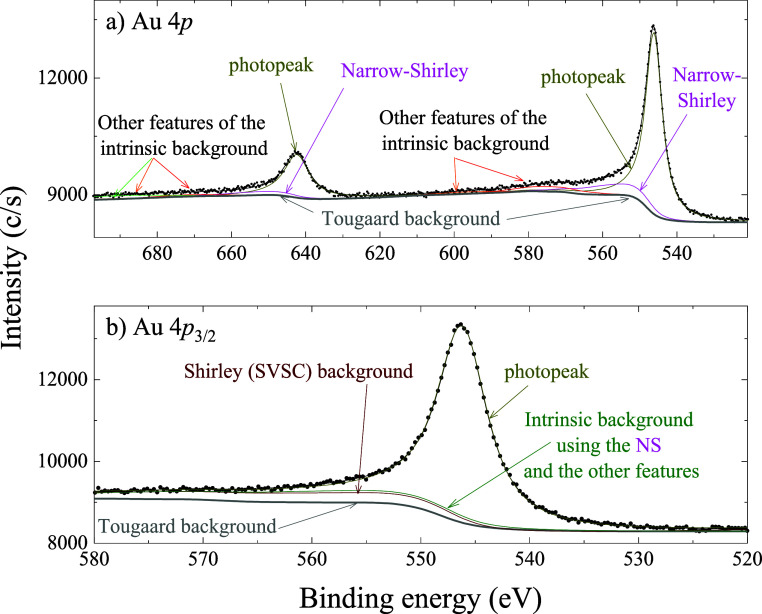
Au 4*p* plotted (a) in a wider range and (b) in the near-peak region. It is possible to appreciate intrinsic background features, including the narrow-Shirley, in the wider region, and the similarity between the Shirley background and the intrinsic background in the near-peak region. The fitting parameters for (a) are shown in Table 2. For the fitting using the SVSC background, the parameters of the photopeak are those of peak p0 in Table 2. The Tougaard background^2^ was computed using an energy loss function of pure gold digitized from ref 26.

**Table 1:** Peak Fitting Parameters of the Sr 3*d* Photoemission Spectrum^a^

line shape	peak	binding energy (eV)	Gaussian width (eV)	Δ*E*_12_ (eV)	Δ*E*_13_ (eV)	Γ_1_ (eV)	Γ_2_ (eV)	Γ_3_ (eV)	*V*_12_ (eV)	*V*_13_ (eV)	*V*_13_ (eV)	area (normalized to the intrinsic photopeaks)
CR III	0	134.3 ± 0.1	0.28 ± 0.11	–0.41 ± 0.72	0.99 ± 0.13	0.00 ± 0.31	2.4 ± 2.0	0.7 ± 0.2	0.69 ± 0.34	0.10 ± 0.10	0.24 ± 0.20	100
				–0.12 ± 0.82	1.25 ± 2.21	–0.27 ± 1.12	4.2 ± 1.0	0.7 (correlated to 3d_5/2_)	1.09 ± 0.63	0.27 ± 0.82	0.60 ± 1.06	
G	1	138.3 ± 0.2	3.4 (fixed)	-	-	-	-	-	-	-	-	30.1 ± 0.2
G	2	142.5 ± 0.1	3.4 (fixed)	-	-	-	-	-	-	-	-	27.5 ± 0.2
G	3	151.8 ± 0.4	6.5 (fixed)	-	-	-	-	-	-	-	-	20.2 ± 0.2
G	4	171.8 ± 0.1	27.7 ± 0.3	-	-	-	-	-	-	-	-	105.0 ± 0.4
G	5	203.8 ± 0.3	20 (fixed)	-	-	-	-	-	-	-	-	14.0 ± 0.4

background parameters				
B-2 (eV^2^)	*s*_NS_ (eV^–1^) (for p0)	*T*_w_ (eV) (for p0)	σ_d_ (eV) (for p0)	baseline (c/s)
3062 ± 5	0.0934 ± 0.0003	10.4 ± 0.4	5.8 ± 1.1	370 ± 1

a p0 (main) and p1 (satellite) correspond to photoemission peaks; the rest of the peaks correspond to the intrinsic background. The spin–orbit splitting of the rest of the peaks was set to the energy difference of the main resonance of the main peak,^20^ which is 1.78 eV. The branching ratio of all the peaks was fixed at 0.6889. The divided cells correspond to the 5/2 (upper) and 3/2 (lower) spin–orbit branches. The resulting eigenvalues of the Hamiltonian are indicated. CR II stands for Type II Coupled-Resonances and G for Gaussian.

**Table 2:** Peak Fitting Parameters of the Au 4*p* Photoemission Spectrum^a^

peak	line shape	4*p*_3/2_ binding energy (eV)	spin–orbit splitting (eV)	Gaussian width (eV)	Lorentzian width (eV)	*s*_NS_ (eV^–1^)	area (normalized to the intrinsic photopeaks)
0	Voigt	546.06 ± 0.02	96.2 ± 0.05	1.6 ± 0.2	4.8 ± 0.1	0.008 ± 0.0001	100
					7.7 ± 0.2		
1	Gaussian	574.67 ± 3.1	correlated to p0	22 (fixed)	-	-	8.0 ± 0.6
2	Gaussian	596.3 ± 3.8	correlated to p0	22 (fixed)	-	-	3.0 ± 0.6
3	Gaussian	690.6 ± 2.1	correlated to p0	30 (fixed)	-	-	6.6 ± 0.6

background parameters		
*T*_w_ (eV)	σ_d_ (eV)	baseline (c/s)
7.1 ± 3.2	13.8 (fixed)	8291 ± 4

a p0 corresponds to a photoemission peak; the rest of the peaks correspond to the intrinsic background. The branching ratio of all the peaks was fixed at 0.3633. The divided cells correspond to the 3/2 (upper) and 1/2 (lower) spin–orbit branches. The total background was modeled with a Tougaard background using an energy loss function for gold for a primary electron energy of 1 keV digitized from ref 26 and a Narrow Shirley background (only applied to peak 0).

## 4 Results and Discussion

### 4.1 Application Examples

The utility of the NS method for revealing the extended intrinsic background structure is demonstrated for various metallic elements. Figure 1c shows the application to a fourth-row metal (Sr 3*d*), Figure 5 to a sixth-row element (Pb 4*f*), and Figure 6 to a fourth-row element (Sc 2*p*). In each case, the NS function models the primary step, while additional peaks are required to fit the remaining structured intrinsic background.

**Figure 5:**
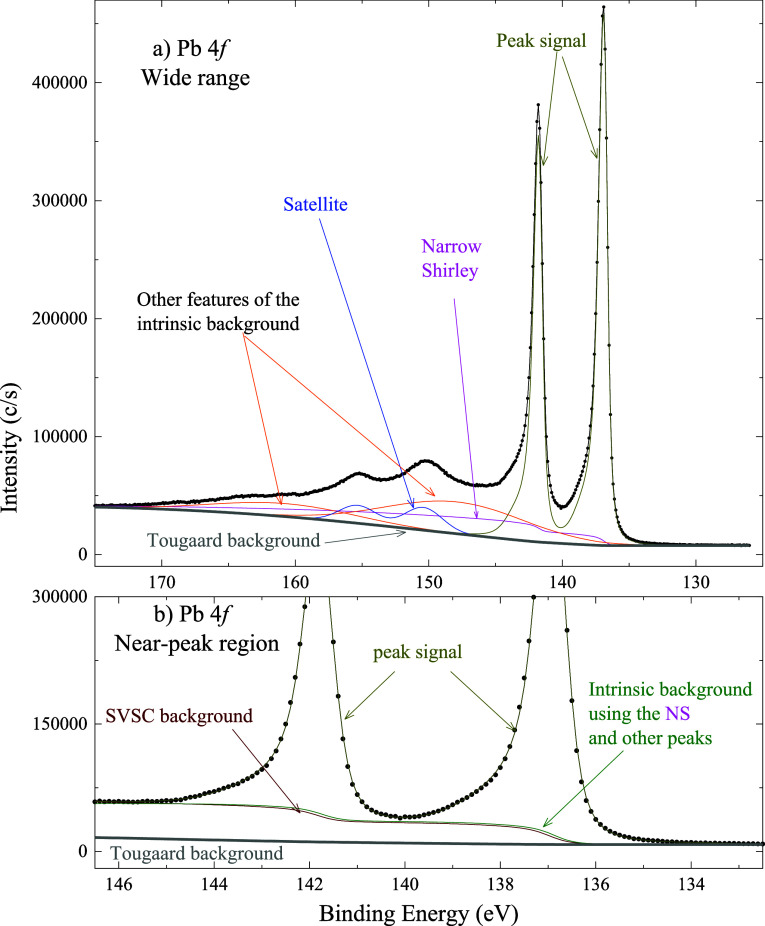
Pb 4*f* spectrum (a) in a wide energy range and (b) in a narrower region typically used for peak-fitting. The intrinsic background can be decomposed into various peaks plus the NS feature. In the narrow region, the intrinsic background modeled with the Shirley algorithm and with the NS plus peaks are similar to each other. The fitting parameters are listed in Table 3.

**Figure 6:**
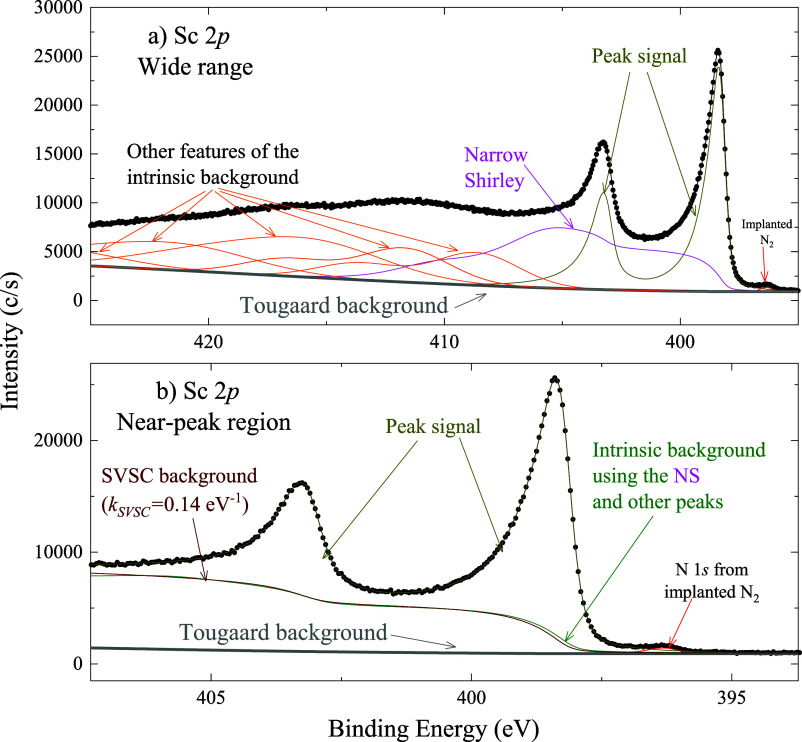
NS applied to 2*p* third-row element Sc. The Sc 2*p* spectrum is shown in (a) a wide energy range and (b) an expanded view of the near-peak region. The wide-range plot reveals a complex background structure, including an NS and additional peaks associated with the intrinsic background. In the near-peak region, the Shirley background and the intrinsic background exhibit similarities.

#### 4.1.1 Application to Pb 4*f* Spectra from Metallic Lead

The wide-range photoemission spectrum of Pb 4f exhibits a satellite in the 145–158 eV BE region (Figure 5a). Reproducing the intrinsic background requires, in addition to the NS signal, two broad Gaussian peaks. The main Pb 4*f* photoemission peak is modeled using asymmetric CR Type III lineshapes, and the satellite using a Gaussian doublet. The fitting parameters are given in Table 3.

**Table 3:** Peak Fitting Parameters of the Pb 4*f* Photoemission Spectrum^a^

peak	lineshape	4*f*_7/2_ binding energy (eV)	Gaussian width (eV)	Δ*E*_12_ (eV)	Δ*E*_13_ (eV)	Γ_1_ (eV)	Γ_2_ (eV)	Γ_3_ (eV)	*V*_12_ (eV)	*V*_13_ (eV)	*V*_23_ (eV)	area (normalized to the intrinsic photopeaks)
p0–4f_7/2_	CR III	137.22 ± 0.01	0.22 ± 0.02	–1.34 ± 0.01	0.24 ± 0.02	0.15 ± 0.01	2.76 ± 0.05	0.74 ± 0.04	–0.71 ± 0.03	0.34 ± 0.02	0.42 ± 0.05	100
p0–4f_5/2_	CR III	142.10 ± 0.01	correlated to p0-4f_7/2_	–1.43 ± 0.01	0.30 ± 0.02	0.20 ± 0.04	3.01 ± 0.05	0.64 ± 1.4	–0.75 ± 0.03	0.33 ± 0.05	0.55 ± 0.01	
satellite	Gaussian doublet	145.72 ± 0.09	3.36 ± 0.24	-	-	-	-	-	-	-		12.9 ± 0.1
p2	Gaussian doublet	158.70 ± 0.03	10.28 ± 0.15	-	-	-	-	-	-	-		38.2 ± 0.1
p3	Gaussian doublet	150.41 ± 0.01	correlated to p2	-	-	-	-	-	-	-		15.05 ± 0.03

background parameters				
B-2 (eV^2^)	C-2 (eV^2^)	*s*_NS_ (eV^–1^) (for p0)	*T*_w_ (eV) (for p0)	σ_d_ (eV) (for p0)
3200 (fixed)	1643 (fixed)	0.015 ± 0.001	21 (fixed)	20 (fixed)

a p0 corresponds to a photoemission peak; the rest of the peaks correspond to the intrinsic background. The spin–orbit splitting of all the peaks was correlated to that of the main peak 4.88 ± 0.11 eV. The branching ratio of all the peaks was fixed at 0.7863. The divided cells correspond to the 7/2 (upper) and 5/2 (lower) spin–orbit branches. CR III stands for coupled-resonances type III.

#### 4.1.2 Application to Sc 2*p* Spectra from Metallic Scandium

The application of the narrow-Shirley (NS) feature to the fit of the Sc 2*p* spectrum from metallic scandium reveals the rich structure of the intrinsic background. As shown in the wide-range spectrum (Figure 6a), the intrinsic background is well reproduced by combining the NS background and a set of broad Gaussian peaks. In the near-peak region (Figure 6b), the intrinsic background (i.e., the sum of the NS structure and the other features) closely resembles the traditional Shirley background. The full peak parameters, presented in Table 4, confirm the suitability of the combined NS and CR approaches for describing metallic scandium.

**Table 4:** Peak Fitting Parameters of the Sc 2*p* Photoemission Spectrum^a^

line shape	peak	binding energy (eV)	gaussian width (eV)	Δ*E*_12_ (eV)	Δ*E*_13_ (eV)	Γ_1_ (eV)	Γ_2_ (eV)	Γ_3_ (eV)	*V*_12_ (eV)	*V*_13_ (eV)	*V*_23_ (eV)	area (normalized to the intrinsic photopeaks)
CR III	p0	398.84 ± 0.11	0.24 ± 0.04	0.13 ± 0.32	–1.01 ± 0.40	0.30 ± 0.13	–0.08 ± 0.70	6.00 ± 1.22	–0.53 ± 0.09	0.68 ± 0.39	1.59 ± 0.31	100.00
CR III		403.85 ± 0.59	correlated to p0	–1.34 ± 1.98	1.09 ± 0.63	–1.94 ± 2.89	13. ± 12	1.15 ± 0.31	–3.33 ± 3.12	0.11 ± 0.11	0.72 ± 0.16	
G	p1	408.63 ± 0.90	4.36 ± 1.16	-	-	-	-	-	-	-		49.0 ± 0.3
G	p2	411.69 ± 0.90	4.15 ± 2.00	-	-	-	-	-	-	-		53.4 ± 0.4
G	p3	415.51 ± 0.80	7.15 ± 4.00									87.0 ± 0.5
G	p4	421.07 ± 0.90	correlated to p3									60.9 ± 0.5
G	p5	426.99 ± 1.61	correlated to p3	-	-	-	-	-	-	-		40.9 ± 0.9
G	N	396.33 ± 0.01	0.63 ± 0.4									0.61 ± 0.02

background parameters					
B-2 (eV^2^)	C-2 (eV^2^)	*s*_NS_ (eV^–1^) (for p0)	*T*_w_ (eV) (for p0)	σ_d_ (eV) (for p0)	baseline (c/s)
3000 (fixed)	1643 (fixed)	0.116 ± 0.001	6.53 ± 0.28	2.18 ± 0.73	911.73 ± 2.05

a p0 corresponds to the photoemission peak; the rest of the peaks correspond to the intrinsic background. The spin–orbit splitting of all the peaks was correlated to that of the main peak 5.01 ± 0.60 eV. The branching ratio of all the peaks was fixed at 0.515. The divided cells correspond to the 3/2 (upper) and 1/2 (lower) spin–orbit branches. CR III stands for Coupled-Resonances type III, and G for Gaussian.

### 4.2 Insights about the Physical Origin of the Intrinsic Background

The previous analysis reveals that the intrinsic background is a substantial component of the photoemission signal, possessing a complex structure extending far beyond the main peak. Despite its significance, its physical origin is frequently overlooked or misinterpreted in the literature. A discussion of the physical mechanisms is therefore essential to this work precisely because the origin of this signal is not well-established and common assumptions (e.g., attributing it solely to extrinsic losses) are inadequate. This section first addresses prevalent misconceptions about the widely used Shirley background (which constitutes the near-peak part of the intrinsic background) and then elaborates on the proposed interchannel coupling with valence band losses (ICVBL) mechanism as a potential origin for the entire extended intrinsic background structure.

#### 4.2.1 Misconceptions about the Shirley Background

The Shirley background algorithm^5^ is popular due to its ability to empirically reproduce the step-like feature near many photoelectron peaks (Figure 1a). However, their physical interpretation is often flawed. It is commonly attributed to photoelectrons that have undergone inelastic scattering, but this interpretation is inconsistent with several key aspects of the photoemission process:^4^

- Extrinsic losses accounted for: all energy losses experienced by photoelectrons after emission (extrinsic losses) are comprehensively described by frameworks like the Tougaard model, which utilizes the electron energy loss function (EELF).^2^ There is no remaining contribution from extrinsic inelastic scattering to be separately assigned to the Shirley background.
- Inclusion leads to incorrect composition: if the intrinsic background signal (including the Shirley component) was treated as part of the primary photoelectron peak signal (i.e., arising from the same initial photoemission event but with subsequent loss), the resulting quantitative elemental compositions would be physically incorrect.^4,27–29^ This indicates it originates from a different process or pathway.
- EELF formalism inapplicable: the EELF formalism, designed for extrinsic scattering where the electron source is effectively delocalized, is unsuitable for describing intrinsic loss events simultaneously occurring with or immediately following the core hole creation at a specific atomic site. This is because the EELF treats all electrons equally, regardless of their scattering history, which is inappropriate for intrinsic events, where the probability of scattering depends on the initial state of the electron and its proximity to the photoemission site. Furthermore, applying the EELF to intrinsic processes leads to inconsistencies, such as an ill-defined electron mean free path.

Therefore, the Shirley background and the broader intrinsic background of which it is a part must originate from intrinsic processes coupled to the photoemission event itself rather than subsequent extrinsic scattering. Furthermore, simplistic explanations fail to account for observed variations in background shape and intensity for different core levels within the same material (e.g., Ni 3*s* vs Ni 3*p* in Figure 7 or Cr 3*s* vs Cr 3*p* in Figure 1 of ref 4), suggesting a mechanism sensitive to the specific electronic structure involved.

**Figure 7:**
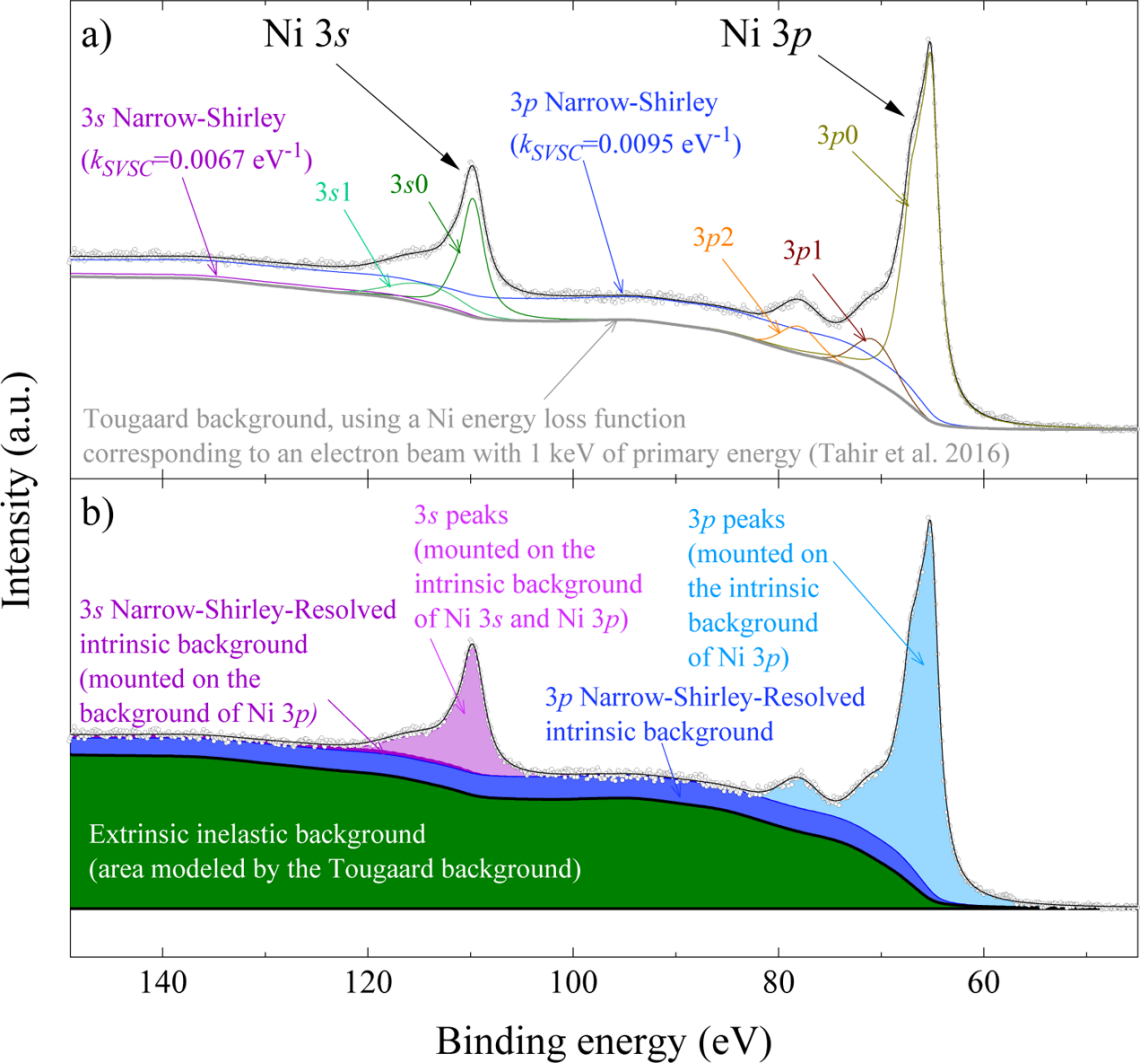
Photoemission spectrum with the regions of Ni 3*s* and Ni 3*p* core levels from a thick film of metallic nickel obtained with a monochromatic Al Kα X-ray source. (a) Fit using the narrow Shirley method encompassed in the software AAnalyzer.^18^ (b) Comparison of the intrinsic signals of Ni 3*p* (blue areas) and Ni 3*s* (light green areas) along with the extrinsic signal for the whole spectrum (green area). The fitting parameters are provided in Table 5.

The Ni 3*p* and 3*s* spectra shown in Figure 7 were fitted simultaneously, with fitting parameters listed in Table 5. The asymmetry of the main peaks was modeled using Coupled-Resonances (CR) type II lineshapes. No oxide peaks were identified. The extrinsic inelastic background was modeled using the Tougaard background method with an energy loss function digitized from ref 30, which corresponds to a thin nickel film and a 1 keV eV primary electron beam.

**Table 5:** Peak Fitting Parameters of the Ni 3*p* and 3*s* Photoemission Spectrum^a^

line shape	peak	3*p*_3/2_ binding energy (eV)	Gaussian width (eV)	Δ*E*_12_ (eV)	Γ_1_ (eV)	Γ_2_ (eV)	*V*_12_ (eV)	area (normalized to the intrinsic photopeaks)
CR II	3p0	65.61 ± 0.04	0.22 ± 0.24	–0.99 ± 0.13	1.75 ± 0.05	1.5 ± 0.4	0.87 ± 0.05	100
CR II				–1.06 ± 0.24	correlated to the 3p_3/2_ branch	2.0 ± 0.8	0.87 ± 0.18	
CR II	3s0	109.9 ± 0.1	1.13 ± 0.33	–1.46 ± 0.20	2.1 ± 0.3	0.7 ± 1.8	0.44 ± 0.20	100
line shape	peak	3*s* binding energy (eV)	Gaussian width (eV)	area (normalized to the respective intrinsic photopeaks)				
G	3p1	70.2 ± 0.2	4.8 ± 0.1	12.5 ± 0.2				
G	3p2	77.6 ± 0.1	Correlated to 3p1	7.6 ± 0.2				
G	3s1	114.2 ± 1.0	8.6 ± 1.5	36 ± 1 (normalized to 3s0)				

background parameters				
*s*_NS_ (eV^–1^) (for 3p0)	*s*_NS_ (eV^–1^) (for 3s0)	*T*_w_ (eV) (3p0 and 3s0)	σ_d_ (eV) (for 3p0 and 3s0)	baseline (c/s)
0.0095 ± 0.0001	0.0067 ± 0.0002	48.3 ± 7.7	55.5 ± 8	83.4 ± 0.4

a p0 (main), p1, and p2 (satellites) correspond to photoemission peaks; the rest of the peaks correspond to the intrinsic background. The spin–orbit splitting of all the 3*p* peaks was correlated to the main peak; the optimal value is 1.04 ± 0.06 eV. The branching ratio of all the 3*p* peaks was fixed at 0.5184. The divided cells correspond to the 3/2 (upper) and 1/2 (lower) spin–orbit branches. The resulting eigenvalues of the Hamiltonian are indicated. CR II stands for type II coupled-resonances and G for Gaussian.

It is worth noting that the extrinsic inelastic background exhibits a subtle structure and successfully reproduces the broad feature observed at around 86–100 eV in the Ni 3*p* region. While this background is notably prominent, it is crucial to recognize that it does not account for the entire area below the photoemission peaks (3p0, 3p1, 3p2, 3s0, and 3s1 in Figure 7a). Peaks 3p1, 3p2, and 3s1 have been associated with satellites.^31^ To fully reproduce the experimental data, an NS background was also required for both Ni 3*p* and Ni 3*s*, with the same width and decay but different associated parameters *s*_NS_.

The behavior of the intrinsic component was noteworthy: the Ni 3*p* spectrum required an NS background that extended well beyond the Ni 3*s* region to the point where its full decay was no longer visible within the analyzed range. In contrast, the Ni 3*s* spectrum required a much weaker NS background, clearly highlighting the core-level dependence of the intrinsic energy loss contribution.

#### 4.2.2 The Proposed Physical Mechanism: Interchannel Coupling with Valence Band Losses (ICVBL)

Building on previous work,^4^ we propose that the interchannel coupling with valence band losses (ICVBL) mechanism is responsible not only for the near-peak Shirley background but for the entire structured intrinsic background revealed by the NS analysis across the extended energy range.

Interchannel coupling itself is a known phenomenon where different photoionization channels interfere.^32,33^ The ICVBL mechanism, depicted schematically in Figure 8d, extends this concept:

- Photon absorption and intermediate state: an incoming photon polarizes a core electron (the “participating” core level) to an intermediate virtual state (Figure 8d uses a 2*p* level as an example) (excitation to a virtual state can be conceptualized as a transient polarization).
- Energy transfer: instead of direct emission, energy is transferred (via Coulomb interaction) to another electron (e.g., a 3*p* electron in Figure 8d) destined for emission.
- Simultaneous valence band loss: during this energy transfer, a portion of the energy excites a valence band electron into the conduction band (represented by the electron–hole pair creation in Figure 8d).
- Electron emission: the second electron is then ejected, but with kinetic energy reduced by the amount lost to the valence band excitation.

**Figure 8:**
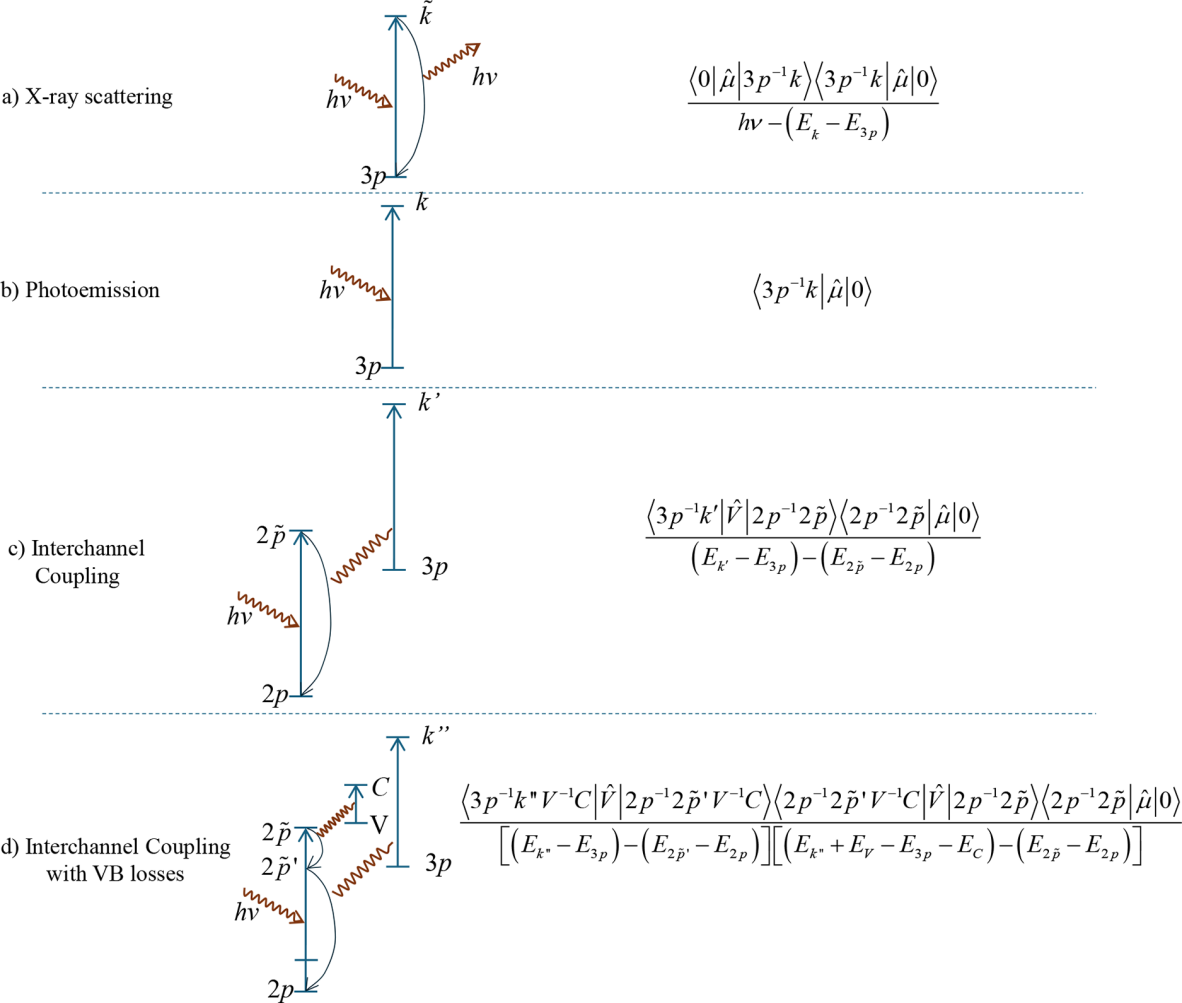
Examples of X-ray-matter interactions showing a relevant matrix element. (a) X-ray scattering is the most common because every electron in the solid interacts with the electromagnetic field of the photon, which could be considered an absorption–reemission process. A matrix element is exemplified with a 3*p* level as an intermediate state. (b) In the photoemission process, the electron keeps the photon energy and escapes. (c) Interchannel coupling is another path for granting the full photon energy to an electron; it interferes with the direct photoemission process of (b). A relevant (second-order) matrix element is exemplified with a 2*p* level as an intermediate state. (d) Interchannel Coupling with valence band losses (ICVBL) is the mechanism proposed for the physical origin of the Shirley signal; this diagram is equivalent to Figure 3 of ref 4. A relevant (third-order) matrix element is exemplified with 2*p*, 2*p̃*, and $2{\mathrm{p̃}}^{\prime }$ levels as intermediate states.

This process results in emitted electrons contributing to the background region at binding energies higher than those of the main photoelectron peak. Because ICVBL leads to the same final state as direct photoemission (a photoelectron and a core hole), the pathways interfere.^24^ The structure observed in the intrinsic background (Figure 1c), including the main NS feature and additional peaks, is proposed to correspond to ICVBL electrons, where specific energy amounts (potentially resonant) are lost to valence band excitations, possibly linked to features in the conduction band density of states.

### 4.3 Predictions of the ICVBL-Intrinsic Background Hypothesis

The hypothesis that the physical origin of the intrinsic background is the ICVBL mechanism connects several seemingly unrelated phenomena. Three of these predictions are discussed in this section.

#### 4.3.1 Modulation of the Intrinsic Background with Photon Energy

The ICVBL mechanism involves the absorption of a photon by a participating deeper core level (e.g., the 2*p* level in Figure 8d). Consequently, the intensity of the intrinsic background associated with the shallower core level (e.g., the 3*p* level in Figure 8d) is expected to be modulated by the photoelectric cross-section of this deeper, participating level. Since this cross-section is strongly modulated in its absorption threshold, the intrinsic background of the shallower level should also be modulated. This predicted behavior, which is distinct from simple absorption effects on the main photopeak, has been experimentally confirmed, such as for the Cr 3*p* Shirley background near the Cr 2*p* edge.^4^

Figure 9 plots the area of the Ti 2*p* narrow-Shirley background normalized to the photopeak area as a function of the photon energy. A noticeable increase in the normalized background area is observed as the photon energy surpasses the Ti 1*s* absorption threshold (∼4966 eV). This modulation is unexpected under any other circumstances and is uniquely explained by the ICVBL mechanism: above the threshold, Ti 1*s* electrons begin to participate in the ICVBL processes that generate the Ti 2*p* intrinsic background. A full description of these experiments is presented in detail in ref 34. The nonzero background area observed below the Ti 1*s* threshold suggests that the Ti 2*p* intrinsic background also arises from ICVBL processes involving other core levels (e.g., Ti 2*s*, 3*s*, 3*p*).

**Figure 9:**
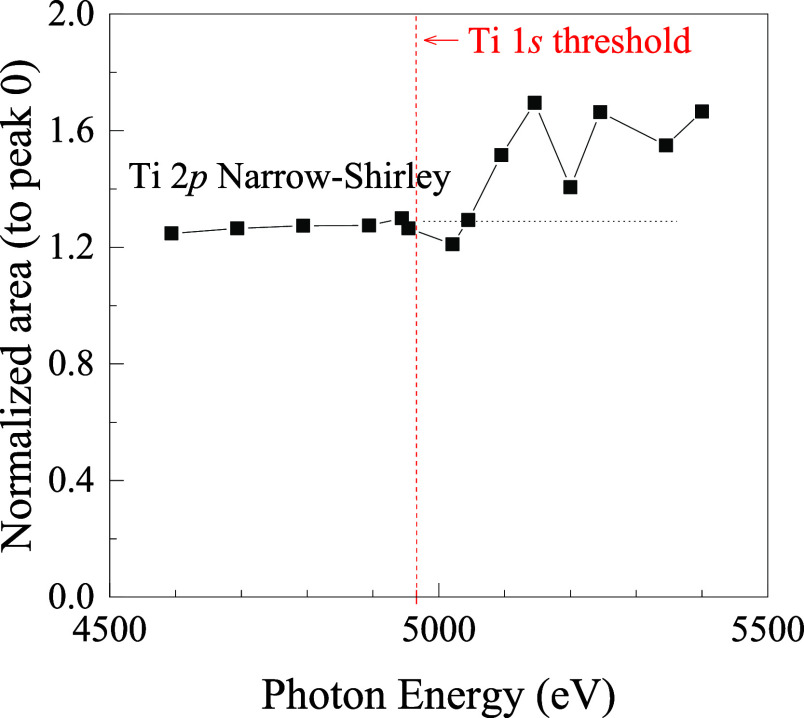
Normalized Ti 2*p* intrinsic background area as a function of photon energy (*h*ν). The area of the Ti 2*p* narrow-Shirley background is normalized to the area of the Ti 2*p* photopeak. A distinct increase is observed as the photon energy crosses the Ti 1*s* absorption threshold (∼4966 eV), providing evidence for the intrinsic core–valence-band-level (ICVBL) mechanism involving the Ti 1*s* core level as the participating core level.

#### 4.3.2 Qualitative Correlation between Intrinsic Background Features and NEXAFS

The ICVBL mechanism posits that the structured features observed in the intrinsic background originate from the excitation of valence-band electrons into the conduction band’s unoccupied states (Figure 8d). Since Near-Edge X-ray Absorption Fine Structure (NEXAFS) also probes the unoccupied density of states (DOS), albeit modulated by different transition matrix elements, the ICVBL hypothesis predicts a potential qualitative correlation between the energy positions of features in the intrinsic background (revealed by NS analysis) and features observed in NEXAFS spectra (Figures 9 and 10). This comparison aims solely to explore potential similarities in the spectral feature positions. The underlying principle is that both ICVBL and NEXAFS mechanisms involve the excitation of electrons into unoccupied conduction band states; in addition to matrix elements, the NEXAFS signal increases with the density of states of the conduction band. In ICVBL, the kinetic energy of the ejected photoelectron is reduced by an amount equal to the energy transferred to excite a valence-band electron. Therefore, as for NEXAFS, regions with a higher DOS in the conduction band should correspond, besides matrix elements, to a larger signal in the intrinsic background.

**Figure 10:**
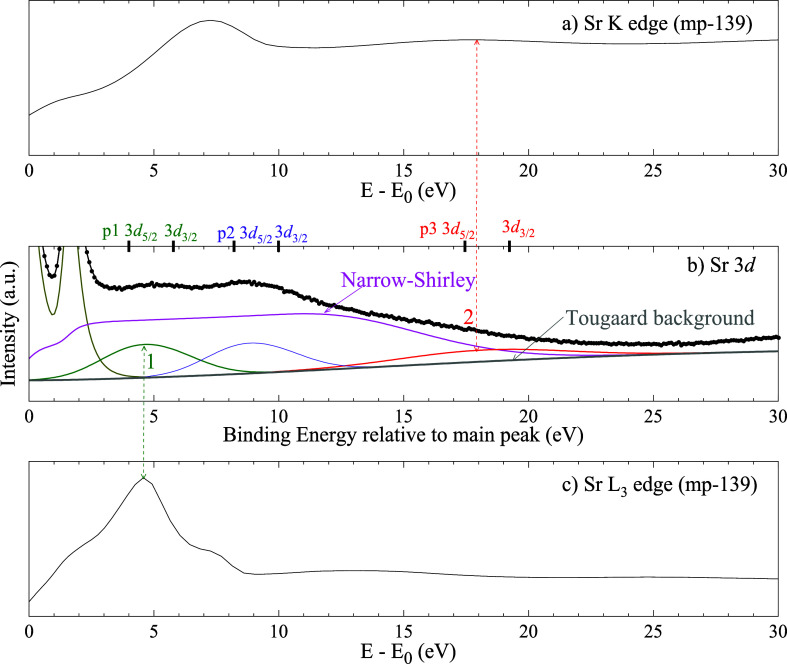
a) Sr K X-ray absorption edge from metallic strontium.^8,9^ (b) Sr 3*d* photoemission spectrum plotted with the binding energy relative to the binding energy (BE) of the main peak and growing to the right. (c) Sr L_3_ X-ray absorption edges from metallic strontium.^8,9^ Besides the narrow Shirley background, the intrinsic background of the Sr 3*d* spectrum exhibits three peaks (p1–p3), two of which correspond to local maxima of the K and L_3_ absorption edges (labeled 1 and 2, respectively). This correspondence, further explored in Figure 8d, suggests a relationship based on the ICVBL mechanism. The positions of the spin–orbit split components of each peak are indicated on the top axis of (b). Fitting parameters for (b) are provided in Table 1

For this qualitative comparison,

- the zero energy for NEXAFS is set at the first-derivative maximum in the edge region (which is a common practice in NEXAFS data analysis), and
- the zero-energy loss for photoemission is set at the energy of the main peak.

The correlation must be interpreted with extreme caution due to numerous factors that complicate a direct quantitative comparison, including differences in matrix elements between ICVBL and NEXAFS, inherent difficulties in precisely defining energy origins in both techniques, a lack of available calculations for the number, positions, and widths of the NEXAFS features, potential differences in sample phases or morphology between XPS and NEXAFS measurements, and the inherent ambiguities in resolving closely spaced peaks within the fitted intrinsic background.

Despite these caveats, frequent alignments between intrinsic background peak positions and NEXAFS features are observed (as indicated by arrows/lines and numerical labels in the figures), lending plausibility to the link via the conduction band DOS suggested by ICVBL. Sr 3*d* and Pd 3*d* are used as examples.

##### 4.3.2.1 Sr 3*d*

The Sr 3*d* photoemission spectrum in Figure 9b corresponds to the data in Figure 1c (the fitting parameters are summarized in Table 1). The main peak was modeled with a Coupled-Resonances type III. The extrinsic background is modeled with the Two-Parameter Universal Inelastic Scattering Approximation) and the intrinsic background was modeled with the NS method.

The intrinsic background shows coincidences at the 17.5 eV feature of the Sr K-edge and at the 4.5 eV feature of the L_3_-edge NEXAFS spectra.

##### 4.3.2.2 Pd 3*d*

The Pd 3d photoemission spectrum of metallic palladium presents a challenging case due to the pronounced asymmetry of the main peak and the presence of sharp features beyond it (Figure 11). Despite this complexity, the full spectrum was successfully modeled. The main peak asymmetry was captured using a coupled-resonance (CR) type III doublet, while the surrounding structure was reproduced using a Narrow-Shirley background combined with symmetric peaks. A detailed analysis found no evidence of oxide-related features. The intrinsic background was modeled with the NS method, and the inelastic background was modeled with the Tougaard method (the energy loss function was obtained from ref 35). The peak-fitting and background parameters are given in Table 6. The 7, 11, 12.5, and 35 eV features of the L_3_-edge coincide with peaks of the intrinsic background; the 13 and 38 eV features of the K-edge coincide with peaks of the intrinsic background.

**Table 6:** Peak Fitting Parameters of the Pd 3*d* Photoemission Spectrum^a^

line shape	peak	3*d*_5/2_ binding energy (eV)	Gaussian width (eV)	Δ*E*_12_ (eV)	Δ*E*_13_(eV)	Γ_1_ (eV)	Γ_2_ (eV)	Γ_3_ (eV)	*V*_12_ (eV)	*V*_13_(eV)	*V*_13_(eV)	area (normalized to the intrinsic photopeaks)
CR III	0	335.54 ± 0.01	0.30 ± 0.01	0.36 ± 0.03	–2.34 ± 0.08	–0.01 ± 0.01	1.17 ± 0.02	6.3 ± 0.3	0.63 ± 0.02	1.23 ± 0.05	–1.05 ± 0.05	100
				–0.51 ± 0.18	–1.5 ± 0.2	0.47 ± 0.04	–0.2 ± 0.5	5.0 ± 0.4	0.71 ± 0.01	0.51 ± 0.11	–1.97 ± 0.23	
G	1	341.31 ± 0.02	1.79 ± 0.07	-	-	-	-	-	-	-		4.7 ± 0.1
G	2	342.7 ± 0.1	5.05 ± 0.20	-	-	-	-	-	-	-		13.0 ± 0.2
G	3	370.3 ± 0.1	10.9 ± 0.4	-								3.6 ± 0.1

background parameters			
*s*_NS_ (eV^–1^) (for p0)	*T*_w_ (eV) (for p0)	σ_d_ (eV) (for p0)	baseline (c/s)
0.0162 ± 0.0001	0.8 (fixed)	19.2 ± 0.3	981 (fixed)

a p0 corresponds to a photoemission peak; the rest of the peaks correspond to the intrinsic background. The branching ratio of all the peaks was fixed at 0.692 and the spin–orbit splitting was correlated to that of the main peak, which is equal to 5.17 ± 0.01. The total background was modeled as a combination of a Tougaard background, a Narrow-Shirley background, and a baseline. The Tougaard background is modeled using an energy loss function derived from pure metallic palladium.^35^ The divided cells correspond to the 5/2 (upper) and 3/2 (lower) spin–orbit branches. The resultant eigenvalues of the Hamiltonian are also reported. CR III stands for Type III Coupled-Resonances and G for Gaussian.

**Figure 11:**
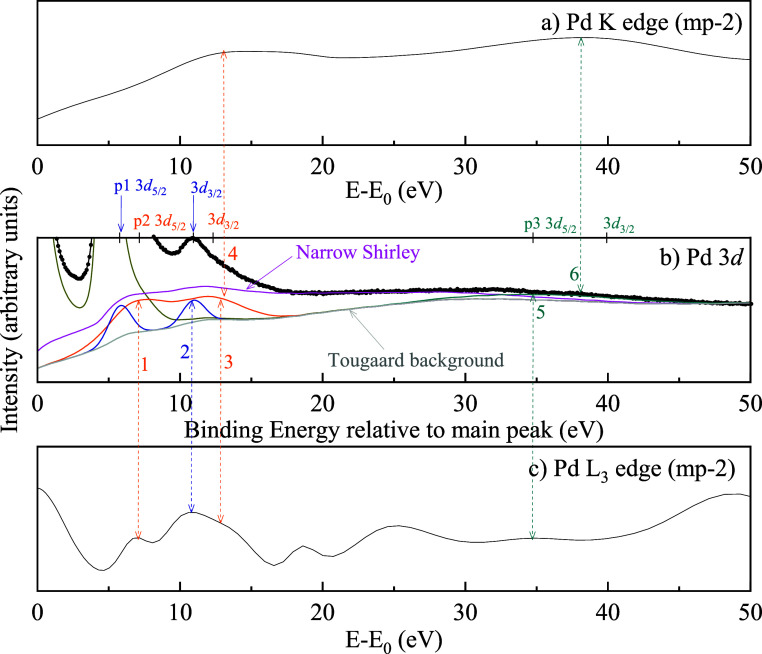
Comparison of (a) NEXAFS spectrum at the Pd K-edge, (b) Pd 3*d* photoemission spectrum, and (c) NEXAFS spectrum at the Pd L_3_-edge. The correspondence between intrinsic background peaks and absorption edge maxima suggests possible relationships between the intrinsic background and regions of high density of states in the conduction band. Fitting parameters for (b) are listed in Table 6.

Additional examples of this type of correlation are provided in ref 25,36 .

#### 4.3.3 Relationship between Intrinsic Background Intensity and Auger Transitions

There are common properties of the ICVBL and Auger mechanisms:

- ICVBL involves energy transfer from a participating core level to the emitted electron’s level, e.g., 2*p*′ → 3*s* transfer (Figure 12c) or 2*p*′ → 3*p* transfer (Figure 12-d). The probability of these transfers influences the intensity of the 3*s* or 3*p* intrinsic background.
- Auger transitions involve a similar energy transfer pathway, e.g., the energy released by 2*p* → 1*s* transition ejects a 3*s* (KL_2,3_M_1_, Figure 12a) or a 3*p* electron (KL_2,3_M_2,3_, Figure 12b).

Therefore, the relative intensities of the intrinsic backgrounds for different core levels (e.g., Ni 3*s* vs Ni 3*p*, Figure 7b) should qualitatively correlate with the relative intensities of the corresponding Auger transitions (e.g., KL_2,3_M_1_ vs KL_2,3_M_2,3_), albeit modulated by different transition matrix elements, allowing only for qualitative comparisons. This is common in all of the cases we studied; here, we present the case of nickel.

Analyzing the Ni 3*s* and 3*p* spectra (Figure 7) reveals a significantly larger intrinsic background (i.e., larger *s*_NS_ parameter, as displayed in Table 5) for 3*p* than for 3*s*; the Ni 3*s*/Ni 3*p* ratio of their associated parameters *s*_NS_(3*s*)/*s*_NS_(3*p*) is 0.70. Therefore, under the ICVBL model, it can be concluded that the probability of energy transfer from 2*p*′ to 3*p* (Figure 12d) is larger than that from 2*p*′ to 3*s* (Figure 12c). This inequality predicts that the KL_2,3_M_1_ transition, which involves energy transfer from the 2*p* → 1*s* transition to 3*s*, should be significantly lower than that for the KL_2,3_M_2,3_ transition, which involves energy transfer from the 2*p* → 1*s* transition to 3*p*.

**Figure 12:**
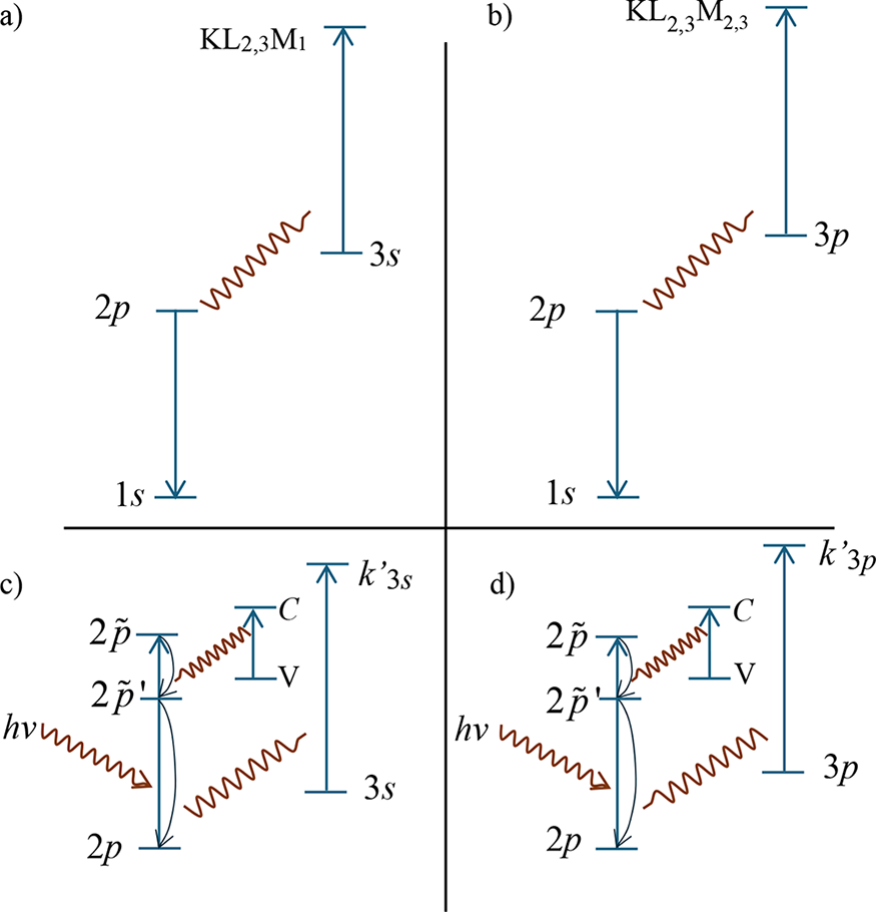
Schematic representation of: (a) a KL_2,3_M_1_ Auger electron transition and (b) a KL_2,3_M_2,3_ Auger electron transition. Panels (c,d) propose interchannel coupling involving valence band excitations as the origin of the intrinsic background observed in the Ni 3*s* and Ni 3*p* spectra (shown in Figure 7). In this model, the energy of a polarized 2*p* electron is transferred to excite a valence-band electron (V to C in the diagrams) and a 3*s* or 3*p* electron. This energy-loss mechanism contributes to the background signal in the corresponding photoelectron spectra.

Examination of the experimental Auger data (Figure 13, Table 7) confirms this trend. Its analysis (discussed below) shows that the KL_2,3_M_1_/KL_2,3_M_2,3_ intensity ratio is 0.65, which is in qualitative agreement with the 0.70 ratio for the intrinsic background intensities mentioned above. This provides further evidence of the ICVBL mechanism as the physical origin of the intrinsic background. Similar analyses are presented for Ti in ref 34.

**Figure 13:**
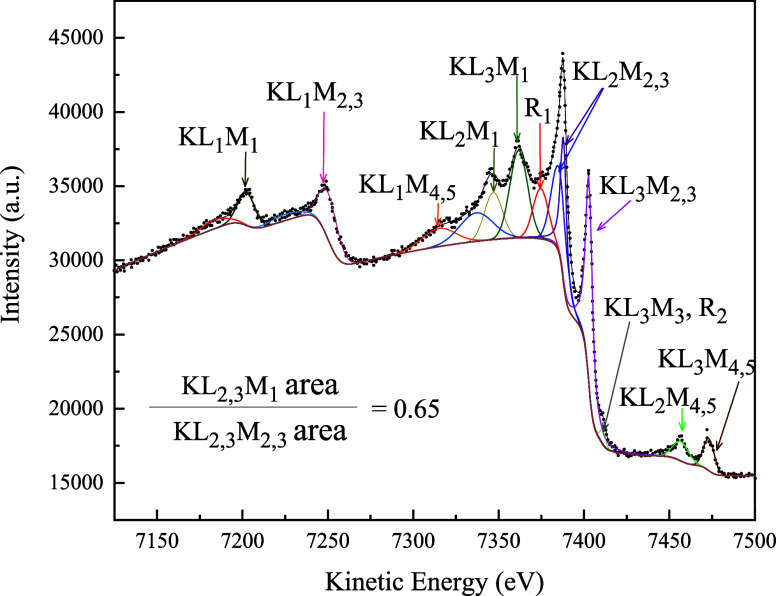
KLM Auger spectra from a nickel metallic film, obtained from ref 14. The details about the peak fitting of the Auger spectra are discussed in the text, and the peak-fitting parameters are displayed in Table 7.

**Table 7:** Peak-Fitting Parameters of the KLM Auger Spectrum Are Displayed in Figure 13 ^a^

kinetic energy (eV)	Gaussian width (eV)	Lorentzian width (eV)	s_*SVSC*_ (eV^–1^)	Auger transition, from refs 14,37	intensity normalized to the sum of the area of the KL_3_M_2,3_ and KL_2_M_2,3_ Auger peaks
7473.1	7.4	0 (fixed)	0.038	KL_3_M_4,5_	0.11
7456.6	10.9	0 (fixed)	0.038 (correlated to peak at 7473.1 eV)	KL_2_M_2_, KL_2_M_4,5_	0.10
7411.0	3.8	0 (fixed)	0.096 (correlated to peak at 7403.2 eV)	KL_3_M_3_, R_2_	0.02
7403.2	1.6	3.9	0.096	KL_3_M_2,3_	1
7388.3	1.8	3.1	0.096 (correlated to peak at 7403.2 eV)	KL_2_M_2,3_	
7385.4	10.5	0 (fixed)	0		0.41
7374.6	10.5 (correlated to peak at 7385.4 eV)	0 (fixed)	0	R_1_	0.24
7361.7	12.2 (correlated to peak at 7346.9 eV)	0 (fixed)	0	KL_3_M_1_	0.65
7346.9	12.2	0 (fixed)	0	KL_2_M_1_	
7336.7	22.9 (correlated to peak at 7314.8 eV)	0 (fixed)	0		0.30
7314.8	22.9	0 (fixed)	0	KL_1_M_4,5_	0.19
7250.3	12.9	0 (fixed)	0.096 (correlated to peak at 7403.2 eV)	KL_1_M_2,3_	0.28
7225.4	22 (fixed)	0 (fixed)	0		0.06
7202.3	11.8	0 (fixed)	0.038 (correlated to peak at 7473.1 eV)	KL_1_M_1_	0.18
7183.6	20 (fixed)	0 (fixed)	0		0.08

a The total background was modeled by applying the active background approach, combining a slope background, SVSC backgrounds, and a baseline. The slope parameter was determined to be −1.05 × 10^–5^ eV^–2^.

A Ni KLM spectrum was analyzed to obtain the KL_2,3_M_1_/KL_2,3_M_2,3_ intensity ratio. The fit shown in Figure 13 is intricate and requires state-of-the-art peak-fitting tools. All peaks were modeled using Voigt lineshapes; however, most yielded an optimal Lorentzian width of 0 eV; to improve fitting stability, Gaussian lineshapes were used instead. The corresponding fitting parameters are listed in Table 7. The total background was modeled as a combination of an SVSC^19^ background—applied to the main Auger peaks—and a slope background.^19^ Most peaks were identified as Auger transitions based on reported kinetic energies.^14,37^

## 5 Conclusions

This work aimed to characterize the structure of the intrinsic background component of the X-ray photoemission signal over an extended energy range and explore its physical origin. To achieve this, we employed the empirical Narrow-Shirley (NS) method as an analytical tool. The NS method, based on the SVSC formalism but incorporating a decay function, allows for the quantification of the intrinsic background over wide binding energy ranges, where conventional Shirley algorithms are unsuitable.

The application of the NS method reveals that the intrinsic background, which includes the near-peak Shirley contribution, is a significant signal component extending typically up to ∼60 eV higher in binding energy than the main peak. This extended intrinsic background exhibits a complex structure, comprising a main NS step-like feature and additional distinct peaks.

Strong arguments were presented, indicating that electrons contributing to the intrinsic background (including the Shirley component) do not originate from extrinsic inelastic scattering of the primary photoelectrons; these extrinsic losses are accounted for separately by Tougaard-like formalisms.

We propose that the interchannel coupling with valence band loss (ICVBL) mechanism offers a compelling physical explanation for the origin and structure of the entire observed intrinsic background, extending the mechanism previously suggested only for the near-peak Shirley signal. Several qualitative predictions derived from the ICVBL hypothesis show encouraging agreement with experimental observations, including:

1) The modulation of intrinsic background intensity when photon energy is tuned near the absorption edge of another participating core level.
2) Qualitative correlations between the energy positions of features within the intrinsic background structure and features in corresponding NEXAFS spectra, consistent with a link via the conduction band density of states.
3) Consistent intensity trends between the intrinsic backgrounds associated with different core levels (e.g., 3*s* vs 3*p*) and the relative intensities of corresponding Auger transitions, reflecting underlying energy transfer probabilities.

Most compellingly, the experiment on Ti 2*p* provides a key piece of evidence: The modulation of the normalized Ti 2*p* NS background area with photon energy clearly demonstrates that above the Ti 1*s* absorption threshold, Ti 1*s* electrons can participate in ICVBL processes. This involvement provides a direct, verifiable mechanism for the Ti 1*s* level to contribute to the intrinsic background of the shallower Ti 2*p* core level photoemission signal. The nonzero intrinsic background intensity observed below the Ti 1*s* threshold also supports the involvement of other shallower levels (Ti 2*s*, 3*s*, 3*p*, and the valence band) in generating the Ti 2*p* intrinsic background.

In summary, this study highlights the importance and complexity of the extended intrinsic background in XPS. While the NS method is not primarily intended for routine background subtraction, it can be applied for that purpose. The ICVBL provides a promising framework for quantitative theoretical modeling of the intrinsic background to extract the electronic structure of the material from experimental intrinsic background data.

## List of acronyms

AES: Auger electron spectroscopy
BE: binding energy
CAE: constant analyzer energy
CR: coupled-resonances line shape
EELF: electron energy loss function
HAXPES: hard X-ray photoelectron spectroscopy
ICVBL: interchannel coupling with valence band losses
NEXAFS: near-edge X-ray absorption fine structure
NIST: National Institute of Standards and Technology
NSLS: National Synchrotron Light Source
NS: narrow-Shirley
RCA: Radio Corporation of America
SVSC: Shirley–Vegh–Salvi–Castle background
XAS: X-ray absorption spectroscopy
XPS: X-ray photoelectron spectroscopy
